# Maltreatment and parenting in youth with primary and secondary callous‐unemotional traits: Anxiety matters

**DOI:** 10.1002/jcv2.12266

**Published:** 2024-07-30

**Authors:** Jessica J. Todorov, Gregor Kohls, Ruth Pauli, Jack Rogers, Anka Bernhard, Katharina Ackermann, Nora M. Raschle, Jules R. Dugre, Aranzazu Fernandez‐Rivas, Miguel Angel Gonzalez‐Torres, Amaia Hervas, Areti Smaragdi, Karen Gonzalez, Ágnes Vetró, Dimitris Dikeos, Arne Popma, Christina Stadler, Kerstin Konrad, Christine M. Freitag, Graeme Fairchild, Rory T. Devine, Stephane A. De Brito

**Affiliations:** ^1^ Centre for Human Brain Health School of Psychology University of Birmingham Birmingham UK; ^2^ Department of Child and Adolescent Psychiatry and Psychotherapy Faculty of Medicine Technische Universität Dresden Dresden Germany; ^3^ Institute for Mental Health School of Psychology University of Birmingham Birmingham UK; ^4^ Department of Child and Adolescent Psychiatry, Psychosomatics and Psychotherapy University Hospital Frankfurt Goethe University Frankfurt am Main Germany; ^5^ Jacobs Center for Productive Youth Development University of Zurich Zurich Switzerland; ^6^ Psychiatric Service Basurto University Hospital Bilbao Spain; ^7^ Child and Adolescent Mental Health Service University Hospital Mutua Terrassa Barcelona Spain; ^8^ Child Development Institute Toronto Ontario Canada; ^9^ Department of Psychology Middlesex University London England; ^10^ Child and Adolescent Psychiatry Department Pediatrics and Child Health Center University of Szeged Szeged Hungary; ^11^ Child and Adolescent Unit of the 1st Department of Psychiatry National and Kapodistrian University of Athens Athens Greece; ^12^ Department of Child and Adolescent Psychiatry VU University Medical Center Amsterdam The Netherlands; ^13^ Department of Child and Adolescent Psychiatry Psychiatric University Hospital University of Basel Basel Switzerland; ^14^ Department of Child and Adolescent Psychiatry, Psychosomatics and Psychotherapy Child Neuropsychology Section RWTH Aachen University Aachen Germany; ^15^ JARA‐Brain Institute II Molecular Neuroscience and Neuroimaging RWTH Aachen & Research Centre Juelich Juelich Germany; ^16^ Department of Psychology University of Bath Bath UK; ^17^ Centre for Developmental Science School of Psychology University of Birmingham Birmingham UK; ^18^ Centre for Neurogenetics University of Birmingham Birmingham UK

**Keywords:** anxiety, callous‐unemotional traits, conduct disorder, FemNAT‐CD, maltreatment, parenting, primary and secondary CU traits

## Abstract

**Background:**

Youth with conduct disorder (CD) and high callous‐unemotional (CU) traits are not a homogenous group and can be disaggregated into primary and secondary subgroups. However, there are inconsistencies in defining primary and secondary subgroups, with some studies using anxiety, others using maltreatment and still others using both features to identify subgroups. There is a paucity of work comparing primary and secondary subgroups with typically developing (TD) youth on experiences of maltreatment and parenting as well as a lack of studies investigating sex differences.

**Methods:**

In a large sample of TD youth (*n* = 946, 66% female) and youth with CD (*n* = 885, 60% female), we used latent profile analysis in youth with CD aged between 9 and 18 years to address four aims: (i) to demonstrate how primary and secondary subgroup membership differs when anxiety, maltreatment, or both are used as continuous indicators, (ii) to compare primary and secondary subgroups with TD youth on abuse and neglect measures, and (iii) to compare primary and secondary subgroups with TD youth on parenting experiences, and (iv) to examine whether the results were consistent across sexes.

**Results:**

Anxiety without maltreatment yielded the best fitting and most theoretically interpretable classification of primary and secondary subgroups across both sexes (Bayesian information criterion = 17832.33, Entropy = 0.75, Lo‐Mendell‐Rubin: *p* < 0.01). Compared with TD youth, youth with primary and secondary CU traits experienced greater levels of abuse and neglect (*p* < 0.001, *η* ^2^_*p*_ = 0.04−0.16) and maladaptive parenting practices (*p* < 0.001, *η* ^2^_*p*_ = 0.04−0.13). Youth with primary and secondary CU traits were equally high on levels of abuse, neglect, and maladaptive parenting (all *p* values >0.05).

**Conclusions:**

We provide evidence that anxiety and maltreatment cannot be used interchangeably to identify youth with primary versus secondary CU traits. Anxiey yielded the best fitting and most theoretically interpretable classifications across both sexes. Our results signify the need for researchers and clinicians to adopt a unified approach to defining primary and secondary subgroups of CU traits using anxiety in both sexes.

## Graphical abstract

We investigated latent classes of typically developing youth with subgroups of primary and secondary callous‐unemotional youth. Sex differences were also explored. The groups were then compared on maltreatment histories (abuse and neglect) and experiences of parenting.

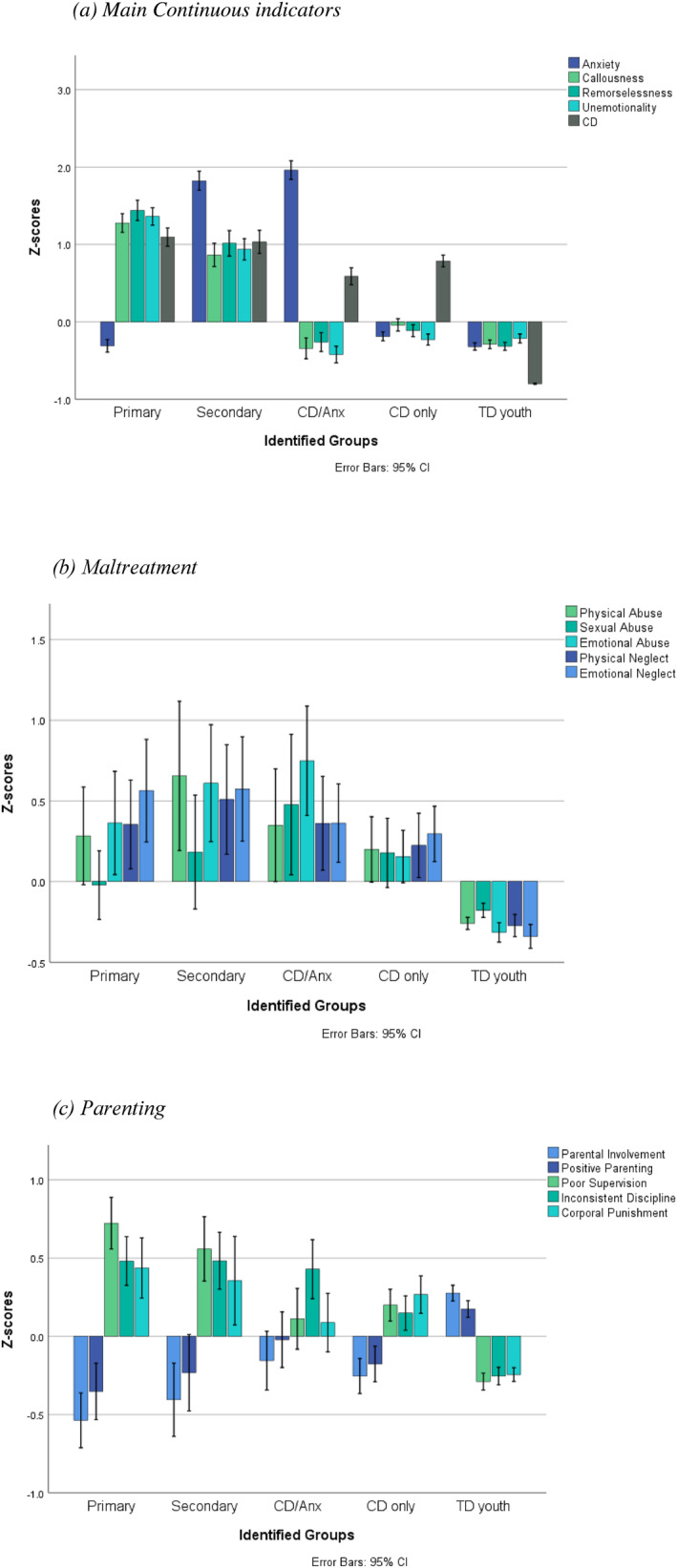

**Key points**

**What's known?**

- Youth with conduct disorder (CD) and high callous‐unemotional (CU) traits are not a homogenous group and can be disaggregated into primary and secondary subgroups.
- There are, however, inconsistencies in defining primary and secondary subgroups, with some studies using anxiety, others using maltreatment and still others using both features to identify subgroups.

**What's new?**

- We provide evidence that anxiety and maltreatment cannot be used interchangeably to identify youth with primary versus secondary CU traits.
- Anxiety yielded the best fitting and most theoretically interpretable classifications across both sexes.
- Youth with primary CU traits and youth with secondary CU traits experienced similar levels of maltreatment but both groups had higher levels of maltreatment than typically developing (TD) youth.
- Youth with primary and secondary CU traits had higher levels of maladaptive parenting than TD youth.

**What's relevant?**

- This work highlights the need for clinical guidelines for identifying primary and secondary CU traits in youth to ensure comparability across studies in the field.
- It is important to include TD youth in research to gain a clearer picture of maltreatment histories and parenting practices in youth with primary and secondary CU traits.

## INTRODUCTION

Conduct disorder (CD) is a psychiatric disorder characterized by aggressive, antisocial behaviour in childhood and adolescence (American Psychiatric Association, 2022; World Health Organization, 2019). The 2019 Global Burden of Disease study conducted across 204 countries found that, among youth aged 0–14 years, CD is the leading cause of burden among psychiatric disorders as measured both in terms of disability‐adjusted life‐years and years lived with disability (Ferrari et al., 2022). Indeed, CD is associated with a broad range of adverse psychosocial outcomes that encompass poor mental health and compromised functioning in family and social relationships and in education and employment (Bevilacqua et al., 2018). CD is a highly heterogeneous disorder with developmental pathways that differ markedly with respect to comorbidity and aetiology (Fairchild et al., 2019). In particular, a subgroup of youth with CD and elevated levels of callous‐unemotional (CU) traits (i.e., lack of remorse and empathy, impaired emotional responding, callousness, uncaring attitudes), the affective component of psychopathy, have been of increasing interest. Indeed, this subgroup is more likely to experience a severe and persistent trajectory of aggression and violence and has increased risk for developing psychopathy in adulthood (Frick et al., 2014b). This subgroup of youth is also characterized by different aetiological risk factors, distinct socio‐cognitive profiles, and require specialized clinical intervention, compared with youth with CD and low levels of CU traits (De Brito et al., 2021; Frick et al., 2014a; Viding & McCrory, 2018). The importance of this high‐risk subgroup of youth has been heralded globally by the addition of CU traits as the "with Limited Prosocial Emotions" specifier for CD in psychiatric classification systems (American Psychiatric Association, 2022; World Health Organization, 2019).

### Primary and secondary subgroups

There is increasing recognition that youth with high CU traits are not a homogenous group and can be further subdivided into low‐anxious primary and high‐anxious secondary subgroups, who may have different aetiological pathways (Fanti et al., 2013; Kimonis et al., 2013; but see Humayun et al., 2014). Historically, according to Karpman (1941), secondary psychopathy in adults developed from experiences of maltreatment while primary psychopathy resulted from genetic factors. However, Karpman (1956) later theorized that primary psychopathy was also influenced by the environment through parental rejection or neglect. Thus, while Karpman (1941, 1956) considered that the high‐anxious secondary subgroup of psychopathy arose from abusive experiences, he did not exclude maltreatment and maladaptive parenting as risk factors for the low‐anxious primary subgroup. Indeed, youth with secondary CU traits may have higher levels of maltreatment than youth with primary CU traits (Fanti et al., 2020), but youth with primary CU traits are equally likely to have experienced maltreatment compared with youth with CD symptoms and low CU traits (Kimonis et al., 2012), and, in some studies, the maltreatment histories (i.e., abuse and neglect in a combined maltreatment measure) of youth with primary and secondary CU traits did not differ (Kimonis et al., 2017; Rosan et al., 2015).

While research on youth with primary and secondary CU traits has increased over the last decade, there are inconsistencies in how these subgroups have been identified (Craig, Goulter, & Moretti, 2021; Todorov et al., 2023). In addition to including CU traits and antisocial behaviour (e.g., either a justice‐involved sample or a measure of antisocial behaviour), studies define primary and secondary subgroups based on either anxiety (Fanti et al., 2013; Kimonis et al., 2017), maltreatment or post‐traumatic stress (Bennett & Kerig, 2014; Fragkaki et al., 2019), or a combination of both anxiety and maltreatment (Fanti et al., 2020; Kimonis et al., 2013). However, phenotypical features (e.g., anxiety) and putative risk factors (e.g., maltreatment) or a combination thereof may not identify the same populations. If youth in the primary subgroup also have maltreatment histories, it is unlikely that maltreatment will differentiate true subgroups of youth with primary and secondary CU traits. As such, inconsistency in how primary and secondary subgroups are identified (i.e., with anxiety or maltreatment) may explain inconsistent findings in the literature. To ensure that researchers and clinicians are studying the same construct and therefore that findings between studies are comparable, it is crucial for the field to adopt a unified approach for classifying youth with primary and secondary CU traits. In line with Karpman's (1941, 1956) view that anxiety is the main phenotypic characteristic distinguishing primary and secondary subgroups, a recent systematic review concluded that anxiety is most used to distinguish variants among youth (Craig, Goulter, & Moretti, 2021).

### Maltreatment

We have argued elsewhere (Todorov et al., 2023) that it is important to distinguish CU trait subgroups using anxiety because it is a symptom whereas maltreatment is a putative causal risk factor that can be present in various ways and produce different outcomes. Indeed, childhood maltreatment is an umbrella term that encompasses types of abuse and neglect (World Health Organization, 2020) and is a hypothesised risk factor for CD and CU traits/psychopathy (de Ruiter et al., 2022; Todorov et al., 2023). However, clear associations between primary and secondary CU traits and maltreatment subtypes have yet to be established. To date, various converging issues have hindered progress in understanding associations between primary and secondary CU traits and maltreatment subtypes. Three of these issues will be discussed in the following section.

The first issue is the inclusion of maltreatment to classify primary and secondary CU traits. A recent meta‐analysis of 29 studies in youth (*N* = 9894) indicated a significant positive association between childhood maltreatment and CU traits, *r* = 0.23, and significant positive associations with physical and emotional abuse and neglect subtypes, *r* = 0.17–0.23 (Todorov et al., 2023). However, associations between maltreatment subtypes and primary and secondary CU traits could not be explored due to inconsistencies in how primary and secondary CU traits were defined accross studies, one of which was the inclusion of maltreatment to define the subgroups. Nevertheless, the meta‐analysis showed that anxiety moderated the strength of association between maltreatment and CU traits, suggesting a distinction between primary and secondary subgroups. More specifically, high anxiety levels were associated with stronger correlations between combined maltreatment (i.e., aggregated abuse and neglect) and CU traits compared with low anxiety. However, it was not possible to investigate whether this was stronger for abuse or neglect subtypes of maltreatment.

The second issue is the practice of collapsing abuse and neglect together into an aggregated maltreatment measure. The high cooccurrence of neglect and abuse (McCrory et al., 2017) has led to the tendency for researchers to collapse them into a broad maltreatment index used to distinguish between those with primary (i.e., low levels of maltreatment) and those with secondary (i.e., high levels of maltreatment). However, this is problematic because the unique effects of abuse and neglect may be obscured. Some forms of abuse and neglect may occur in isolation (e.g., physical or emotional neglect without a form of abuse, or emotional abuse without physical neglect). Findings among children (*N* = 674) with documented abuse (51.6%) suggest that while abuse and neglect commonly cooccur (57% of maltreated children), a significant portion (31%) of maltreated children experience neglect only (Warmingham et al., 2019). Further, abuse (e.g., threat) and neglect (e.g., deprivation) likely influence different developmental processes and outcomes (Dayananda et al., 2023; McLaughlin & Sheridan, 2016). Youth in primary and secondary CU subgroups may share qualitatively different maltreatment histories but similar outcomes (i.e., equifinality). In a recent review of studies examining primary and secondary CU traits (Craig, Goulter, & Moretti, 2021), of the studies that included measures of maltreatment (*k* = 16), studies either measured abuse only or collapsed abuse and neglect together into an overall maltreatment score (*k* = 13) with very few studies examining the maltreatment subtypes separately (*k* = 3). When maltreatment is aggregated into an overall abuse and neglect measure, it is not possible to investigate subtype associations with primary and secondary CU traits (Todorov et al., 2023).

The third issue is that most studies to date that have included maltreatment measures have focussed on incarcerated samples. There is a paucity of studies comparing youth with CU traits to TD youth whilst also including measures of maltreatment. For example, of the studies that included TD youth in the recent review by Craig and colleagues (2021), 12 studies included community samples but none of these included maltreatment measures. The lack of comparison between youth with CU variants and TD youth is problematic because most incarcerated youth are likely to have histories of maltreatment (Moore et al., 2013; Vahl et al., 2016). Thus, studies showing that youth with primary CU traits have experienced similar levels of maltreatment to incarcerated non‐CU trait youth (Euler et al., 2015; Kimonis et al., 2012) may be confounded by the fact that justice‐involved samples are characterised by higher‐than‐average levels of maltreatment. In this context, a strength of the current study is the inclusion of TD youth and measures of maltreatment subtypes. A clearer picture of the severity of maltreatment histories for youth in the primary and secondary subgroups could be obtained by comparing them with TD youth.

Thus, inconsistency in the way subgroups of youth with primary and secondary CU traits are classified, the aggregation of abuse and neglect into one measure of maltreatment, and a lack of TD youth in studies exploring maltreatment are three issues that highlight how the existing literature obscures an understanding of whether the primary subgroup is also associated with a maltreatment history or whether associations with abuse and neglect are stronger in either subgroup of youth with CU traits.

### Parenting practices

In addition to maltreatment, other parenting practices are important to consider because they play a central role in the development and maintenance of CD in general and CD with CU traits in particular (Pauli et al., 2021; Vaughan et al., 2021; Waller et al., 2015) and they are operationalized in similar but distinct ways (Backhaus et al., 2023). While maladaptive parenting and maltreatment tend to be highly correlated, they are not completely overlapping and index distinct parenting practices. CU traits have high genetic risk, but they are likely expressed or suppressed through epigenetic processes that involved a complex interplay between genetic inheritance and environmental factors (Tomlinson et al., 2022). For example, increased parental involvement and warmth can buffer hereditary risk for the development of CU behaviours (Hyde et al., 2016; Waller et al., 2016). Conversely youth with CU traits tend to elicit harsher parenting that may increase rather than decrease the expression of CU traits (Hyde & Dotterer, 2022). Further, treatment for youth with CU traits shows the greatest therapeutic gains when parent training is included (Perlstein et al., 2023).

To date, specific parenting practices that are linked to CU traits in longitudinal research include decreased parental involvement (e.g., having conversations with children about their day, attending school events), low levels of positive parenting (e.g., praising positive behaviour, showing affection), poor supervision (e.g., not monitoring children's whereabouts or activities with friends), inconsistent discipline (e.g., not following through with punishments), and corporal punishment (e.g., slapping, hitting) (Fanti et al., 2017; Hawes et al., 2011). However, it is unclear to what extent, if any, primary and secondary subgroups may differ from each other on experiences of negative parenting (i.e., harsh and inconsistent discipline). Indeed, findings to date in longitudinal research (Bégin et al., 2021; Meehan et al., 2017) and twin studies (Humayun et al., 2014) have suggested equally high experiences of negative parenting and equally low levels of parental warmth (Bégin et al., 2021). Despite growing recognition of the importance of the links between parenting and primary and secondary CU traits (Kimonis, 2023; Perlstein et al., 2023), maladaptive parenting such as decreased parental involvement and poor supervision remain unexplored in primary and secondary subgroups.

### Sex differences

Beyond reporting proportions of males to females within primary and secondary CU subgroups, of the 21 studies to date that included both sexes, nine did not report on sex differences and 12 reported the proportion of males to females only, with inconsistent findings (Craig, Goulter, & Moretti, 2021). Data from an all‐female study suggest that youth with secondary CU traits experienced more negative parenting than youth with primary CU traits (Goulter et al., 2017), however no mixed‐sex study has compared primary and secondary subgroups to TD youth across different subtypes of maltreatment or parenting practices.

#### Present study

The present study had four aims. The first aim was to identify subgroups of youth with primary and secondary CU traits using anxiety, and to establish whether results differ when maltreatment, or both anxiety and maltreatment, are used as distinguishing features alongside CU traits in youth with CD. Using anxiety alone, we predicted that two subgroups of youth with CU traits with differing anxiety levels would be identified and that group allocation would be different when maltreatment was used. The second aim was to compare the experiences of youth in the primary and secondary CU trait subgroups to TD youth on maltreatment subtypes of abuse and neglect. We hypothesized that youth with both primary and secondary CU traits will have been exposed to higher levels of maltreatment than TD youth, but that secondary subgroups may have higher levels of abuse and primary subgroups may have higher levels of neglect compared with TD youth. The third aim was to compare experiences of parenting in youth with primary and secondary CU traits to TD youth. We hypothesized that both primary and secondary subgroups would have higher rates of negative parenting practices and lower rates of positive parenting experiences than TD youth. Due to the paucity of research on parenting practices in relation to primary and secondary CU subgroups, we did not formulate specific predictions about differences between CU subgroups. And finally, the fourth aim was to repeat all analyses in boys and girls separately. Due to the paucity of research on sex differences within primary and secondary subgroups of CU traits, we made no a priori hypotheses regarding sex differences.

## METHOD

### Participants

This study included 885 youth with CD (60% female) and 946 TD youth (66% female), aged between 9 and 18 years (*M*_age_ = 14.10 years, SD = 2.42), from the European FemNAT‐CD project (Freitag et al., 2018). FemNAT‐CD is a multi‐site study with participants recruited from countries across Europe including the UK, Ireland, Germany, Switzerland, Spain, Greece, Hungary, and The Netherlands. Participants were recruited from clinics, schools, youth offending services, and the wider community. Exclusion criteria for CD and TD youth were IQ < 70, autism spectrum disorders, a history of manic or psychotic episodes, neurological or genetic disorders. TD youth were excluded if they had a history of externalizing disorders or any current psychiatric disorders. Socioeconomic status (SES) information was part of FemNAT‐CD standard procedure which included parental income, education level, and occupation and was standardized by country.

### Ethical considerations

Ethical committees at each site approved the study. Ethical approval details for the sites are provided in Supplemental S1.

### Measures

#### Conduct disorder and anxiety

We used the Kiddie Schedule for Affective Disorders and Schizophrenia—Present and Lifetime version which was administered separately to participants and caregivers to assess for CD and anxiety (K‐SADS‐PL; Kaufman et al., 1997) This semi‐structured diagnostic interview assesses current and past psychopathology in children and adolescents and yields reliable diagnoses across a range of symptoms, including affective and anxiety disorders (Kaufman et al., 1997). Interviews were conducted by trained staff at each site with participants and one caregiver as an additional informant. Due to the age range of participants in the study, participants were included in the CD group if they (i) were between 9 and 12 years of age and met DSM‐5 diagnostic criteria for oppositional defiant disorder (ODD) with at least one symptom of CD present, (ii) were over 12 years and met diagnostic criteria for ODD with at least two symptoms of CD present, or (iii) met diagnostic criteria for CD. Most K‐SADS‐PL items were scored using a 0‐to‐3‐point rating scale with scores of 0 indicating no information is available, scores of 1 suggesting the symptom is not present, scores of two indicate subthreshold levels of symptomology, and scores of 3 representing threshold criteria. Total scores were used to create overall ODD and CD categories.

K‐SADS‐PL anxiety disorder subscales were dichotomized (i.e., 0 = symptoms not present, 1 = subthreshold/threshold) and summed to create a total anxiety score. Fifteen items were included from Generalized Anxiety Disorder (e.g., unrealistic worry, marked self‐consciousness, somatic complaints), Social Phobia (e.g., shrinks from contact), Panic Disorder (e.g., dizzy, shortness of breath), and Separation Anxiety (e.g., fear of being alone, fear of harm befalling a loved one). Interrater reliability for K‐SADS‐PL items was high (95% agreement; *κ* = 0.91).

#### CU traits

CU traits were assessed with the self‐report version of the Youth Psychopathic traits Inventory a 50‐item measure of psychopathic traits (Andershed et al., 2002). Each item was answered on a four‐point scale ranging from “does not apply at all” (1) to “applies very well” (4). CU trait scores were calculated using the “callousness” (e.g., “*When other people have problems, it is often their own fault, therefore, one should not help them.*”), “unemotionality” (e.g., “*I usually feel calm, when other people are scared.*”), and “remorselessness” (e.g., “*I have the ability not to feel guilt and regret about things that I think other people would feel guilty about.*”) subscales with good internal consistency (*α* = 0.81−0.93; Supplemental Table S1).

#### Childhood maltreatment

Childhood maltreatment was assessed using the self‐report version of the Childhood Trauma Questionnaire (CTQ; Bernstein & Fink, 1998). The CTQ is a 28‐item questionnaire with five subscales, each containing five items, which measure childhood histories of abuse (i.e., physical, sexual, and emotional abuse) and neglect (i.e., physical and emotional). Items were rated between 1 (*never true*) and 5 (*very often true*). Total scores were used for each CTQ subscale (*α* = 0.82–0.93; Supplemental Table S1). For physical abuse, scores below 7 were considered minimal to moderate and scores between 8 and 12 were considered moderate to severe. For sexual abuse, scores below 5 were considered minimal to moderate and scores between 6 and 12 were considered moderate to severe. For emotional abuse, scores below 8 were considered minimal to moderate and scores between 9 and 15 were considered moderate to severe. For Physical neglect, scores below 7 were considered minimal to moderate and scores between 8 and 12 were considered moderate to severe. For emotional neglect, scores below 9 were considered minimal to moderate and scores between 10 and 17 were considered moderate to severe.

#### Parenting practices

To assess parenting practices, child and parent versions of the Alabama Parenting Questionnaire (APQ; Essau et al., 2006) were used. The APQ is a 42‐item measure of parental involvement (e.g., “You ask your child about his/her day”), positive parenting (e.g., “You offer your child rewards for behaving well”), poor supervision (e.g., “Your child is out with friends you don't know”), inconsistent discipline (e.g., “Your child talks you out of being punished after he/she does something wrong”), and corporal punishment (e.g., “You slap your child when he/she has done something wrong”) with internal consistency ranging from *α* = 0.63–0.90 (Supplemental Table S1). Items were rated on a five‐point scale ranging from 1 (*never*) to 5 (*always*). Consistent with our previous work (e.g., Pauli et al., 2021), The more negative score between parent and child ratings for each item was taken as the item score. That is, for the lower score on the parental involvement and positive parenting items and the higher score on the poor supervision, inconsistent discipline, and corporal punishment items.

#### IQ

To assess IQ, English‐speaking sites used the vocabulary and matrix reasoning subscales of the Wechsler Abbreviated Scale of Intelligence (Wechsler, 1999) Other sites used the vocabulary, block design, and matrix reasoning tests of the Wechsler Intelligence Scale for Children (Wechsler, 2003) for participants under the age of 16 and the Wechsler Adult Intelligence Scale for participants aged 17–18 years (Wechsler, 2008).

### Socioeconomic status

SES was assessed using standard FemNAT‐CD protocol combining parental income, education, and occupation (Pauli et al., 2021). Human and computer‐based ratings were combined into a factor score using principal component analysis with acceptable reliability (*α* = 0.74). To account for economic variation between countries, final SES scores were scaled and mean‐centred within each country to provide a relative measure of SES.

### Analysis

The first aim was to investigate whether latent profiles in youth with CD would differ when anxiety or maltreatment, or both, were used as continuous indicators alongside CU traits. We used Latent Profile Analysis (LPA) in Mplus 8 (Muthén & Muthén, 1998‐2017) within the sample of youth with CD to identify subgroups. We conducted LPA with three distinct combinations of continuous indicators (i) CU traits and anxiety, (ii) CU traits and maltreatment, or (iii) CU traits, anxiety, and maltreatment. The LPAs were conducted in the CD subsample because youth with primary and secondary CU traits exist as subgroups of youth with CD, not within subgroups of TD youth. A goal and novel aspect of this study was to compare these two variants *with* TD youth by first identifying them within the clinical group with CD and then using the TD group as a benchmark with which to compare youth with primary and secondary variants of CU traits.

LPA identifies latent groups by decomposing the covariance matrix, which reveals clusters of relationships between individuals and groups them accordingly (Ferguson et al., 2020). To identify the optimal number of classes to retain, the Bayesian information criterion (BIC) and Akaike information criterion (AIC) were compared for relative fit across and between models (Nylund et al., 2007). The lowest BIC and AIC values were preferred as long as they indicated a solution in which each latent class had a clear theoretical interpretation (Geiser, 2013). We also used the Lo‐Mendell‐Rubin (LMR) statistic, which tested *k* – 1 classes against *k* classes, with a significant chi‐square value (*p* < 0.05) indicating the *k* class model was preferred over the *k* – 1 class model (Lo et al., 2001). Entropy values, which range from zero to one, were used to evaluate the accuracy of classification by how well the data were partitioned into profiles (Ferguson et al., 2020). Values of >0.80 suggested highly discriminated latent profiles (Celeux & Soromenho, 1996; Tein et al., 2013). The models were compared on goodness‐of‐fit indices (i.e., AIC, BIC, SABIC) and configural invariance was assessed using the bootstrap likelihood ratio test, with decreases in model fit indicating latent profiles differed between groups (Finch, 2015). To compare class allocation between the best fitting model from each combination of continuous indicators, *χ* ^2^ analyses were conducted. Additionally, to compare anxiety levels in the best fitting model using maltreatment only, a one‐way ANOVA was conducted.

To investigate whether the models would hold equally well in boys and girls, we followed three steps (Morin et al., 2016). First, we tested the class solutions in boys and girls separately. Second, we tested the configural (unconstrained) model in boys and girls simultaneously. Third, we tested the structural (constrained) model in boys and girls simultaneously. Lower AIC, BIC, and SABIC values indicated the better fitting model (Morin et al., 2016).

To examine subtypes of maltreatment (aim 2) and experiences of parenting (aim 3), differences between LPA classes and TD youth, maltreatment subtypes and parenting practices were examined using multivariate analyses of covariance (MANCOVAs) with Bonferroni‐corrected post‐hoc tests. These steps were completed in the overall sample and within each sex separately (aim 4), while controlling for covariates such as age, IQ, and SES.

## RESULTS

### Latent profile analysis

Descriptive statistics and correlations among variables are reported in the supplemental materials (Supplemental Table S1). LPA models from 2 to 5 classes were compared using the three combinations of continuous indicators (i.e., anxiety, maltreatment, or both) to identify the optimal number of profiles to retain within youth with CD.

For the first combination of continuous indicators (Table 1a) using CU traits and anxiety, the BIC and AIC values decreased across models, but the LMR revealed there was no significant difference in fit between the four and five‐class models. As a result, the more parsimonious four‐class model was retained, which was comprised of two high CU trait subgroups and two CD‐only subgroups (i.e., low CU trait subgroups), each of which had high and low anxiety (entropy = 0.75).

**TABLE 1:** Model fit summaries.

Classes	Log likelihood	AIC	BIC	SABIC	Entropy	Smallest class %	LMR
a) CU traits and anxiety							
2	−8927.7	17881.41	17943.62	17902.34	0.7	31	*p* < 0.001
3	−8881.44	17798.87	17885.01	17827.85	0.73	24	*p* < 0.001
4	−8838.13	17722.26	17832.33	17759.28	0.75	12	*p* < 0.01
5	−8803.99	17663.98	17797.98	17709.05	0.73	5	0.12
b) CU traits and maltreatment							
2	−7601.19	15228.38	15290.43	15249.14	0.71	32	*p* < 0.001
3	−7563.88	15163.76	15249.68	15192.51	0.74	5	0.1
4	−7548.56	15143.13	15252.91	15179.86	0.7	5	0.1
5	−7534.53	15125.05	15258.7	15169.78	0.64	4	0.19
c) CU traits, anxiety, and maltreatment							
2	−9958.48	19948.96	20025.53	19974.71	0.7	32	*p* < 0.001
3	−9910.8	19865.61	19970.89	19901.02	0.73	24	*p* < 0.001
4	−9867.65	19791.29	19925.29	19836.37	0.75	12	*p* < 0.01
5	−9832.3	19732.61	19895.32	19787.34	0.73	6	0.1

Abbreviations: AIC, Akaike information criterion; BIC, Bayesian information criterion; CU, callous unemotional; LMR, Lo‐Mendell‐Rubin; SABIC, Sample‐size Adjutsted Bayesian information criterion.

For the second combination of continuous indicators (Table 1b) using CU traits and maltreatment, the BIC and AIC values decreased across models, but the LMR revealed there was no significant difference in fit between the three, four, and five‐class models which had smallest class counts of <5% and entropy ranging from 0.64 to 0.74. The two‐class model was therefore retained, which had a group of youth with high CU traits and high maltreatment and a group with low CU traits and high maltreatment.

For the third combination of continuous indicators (Table 1c) using CU traits, anxiety, and maltreatment, model fit statistics (i.e., AIC, BIC) were worse than model results for the first combination (i.e., CU traits and anxiety) but with similar entropy and LMR statistics. Because the five‐class model was not significantly different from the four‐class model, the four‐class model was retained which, like the first combination, had subgroups of high CU trait and CD‐only youth who differed significantly on levels of anxiety, but none of the subgroups differed significantly on maltreatment (entropy = 0.75).

Follow up *χ* ^2^ tests were used to assess group allocation differences (Table 2). There was a significant association between the four‐class CU trait/anxiety model and the two‐class CU trait/maltreatment model, *χ* ^2^ (3) = 725.32, *p* < 0.001, *φ* = 0.91. Most children (94.2%) in the low CU/high maltreatment group were classified in the CD‐only (i.e., low CU groups). Likewise, most children (96.7%) in the high CU/high maltreatment group were classified in the primary or secondary groups (i.e., the high CU groups). Thus, the CU/Maltreatment classifications captured differences in levels of CU traits rather than any differences in experiences of maltreatment.

**TABLE 2:** Chi‐sqaure results for maltreatment and anxiety only LPAs.

	CU + maltreatment				
	Low CU, high MT (*n* = 603)		High CU, high MT (*n* = 271)		*χ* ^2^
	*n*	%	*n*	%	
CU + anxiety					
Primary	0	0	195	72	
Secondary	35	6	67	25	
CD/Anx	434	72	9	3	725.32
CD only	134	22	0	0	

Abbreviations: Anx, anxiety; CU, callous unemotional; MT, maltreatment.

The anxiety‐only and anxiety and maltreatment combinations, *χ* ^2^ (9) = 2576.87, *p* < 0.001, *φ* = 1.71, also supported the idea that adding maltreatment to the LPA did not add any further information than when using CU traits and anxiety. Indeed, there was near‐perfect agreement between the two classification approaches in classifying primary (99%) and secondary (100%) children and CD‐only children with high levels of anxiety (96.3%) and CD‐only children with low level of anxiety (99.1%). Thus, including maltreatment as a continuous indicator did not aid classification in the LPA. The remaining study aims were therefore addressed using the first combination based on CU traits and anxiety with four subgroups.

Participants (*n* = 195; 11%) scoring high on CU traits and low on anxiety (46% girls) were designated as the primary CU trait subgroup. Participants (*n* = 102; 6%) scoring high on CU traits and high on anxiety (63% girls) were labelled as the secondary CU trait subgroup. Participants (*n* = 135; 7%) scoring low on CU traits and high on anxiety were designated as the CD/Anx subgroup (74% girls). Participants (*n* = 453, 25%) scoring low on CU traits and low on anxiety were designated as the CD‐only subgroup (62% girls). The remaining participants (i.e., youth who did not meet diagnostic criteria for CD and who scored below threshold on CU traits and were not included in the LPA; *n* = 946, 51%) were designated as TD youth (66% girls).

**FIGURE 1:**
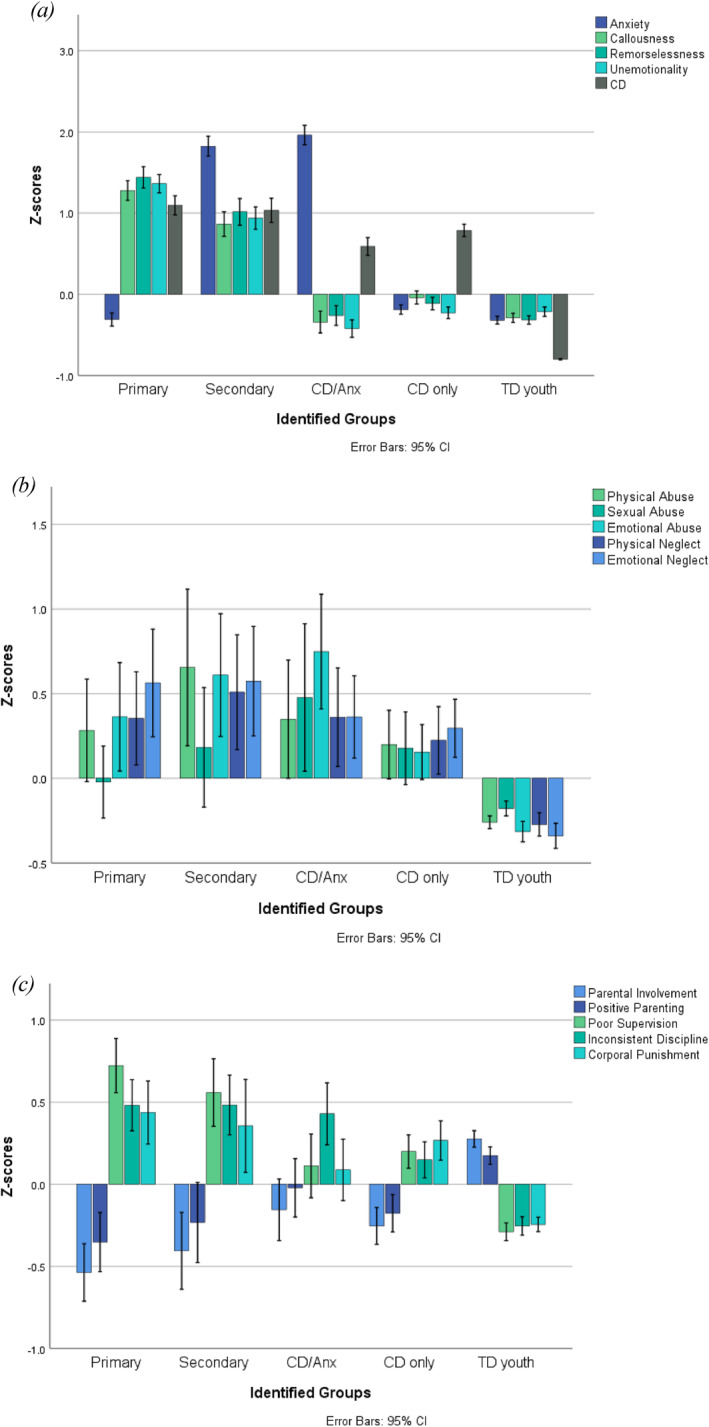
(A) Main continuous indicators (B) maltreatment (C) parenting.

Confirming the distinction between the subgroups, a MANCOVA comparing scores on the continuous indicators was significant, Wilk's *λ* = 0.09, *F* (20, 5988) = 316.99, *p* < 0.001 (Table 3, Figure 1A). Primary and secondary subgroups had significantly higher levels of CU traits than TD and other CD subgroups, and the primary subgroup had significantly higher levels than the secondary subgroup. Youth in the high CU trait (i.e., primary and secondary) and low CU trait subgroups (i.e., CD/Anx and CD‐only) had significantly higher levels of CD than TD youth, while primary and secondary subgroups had significantly higher levels of CD than CD/Anx and CD‐only youth. Primary and secondary subgroups did not differ from each other on levels of CD.

**TABLE 3:** Comparisons between identified groups.

Variable	Total	Primary	Secondary	CD/Anx	CD‐only	TD youth	*f/X* ^2^	*p*	$\eta_{p}^{2}$
	*N* = 1831	*N* = 195	*N* = 102	*N* = 135	*N* = 453	*N* = 946			
Age	14.09 (2.42)	14.53 (2.28)^a^	14.09 (2.25)^a^	13.99 (2.35)^a^	14.03 (2.35)^a^	14.05 (2.49)^a^	1.5	*p* = 0.2	0.01
Sex (% female)	64%	46%	63%	74%	62%	66%	36.17	*p* < 0.001	
IQ	99.67 (14.07)	94.8 (12.23)^a^	95.04 (14.36)^a^	94.84 (14.37)^a^	94.4 (14.15)^a^	103.79 (12.92)^b^	47.26	*p* < 0.001	0.1
SES	0.01 (1.01)	−0.38 (1.05)^a^	−0.24 (0.95)^a^	−0.51 (0.93)^a^	0.35 (0.98)^a^	0.31 (0.92)^b^	52.76	*p* < 0.001	0.12
Classification variables									
CD	2.65 (3.2)	6.17 (2.66)^a^	5.97 (2.44)^a^	4.54 (2.03)^b^	5.18 (2.58)^c^	0.09 (0.33)^d^	1056.07	*p* < 0.001	0.7
Callousness	10.09 (3.01)	13.94 (2.56)^a^	12.7 (2.31)^b^	9.06 (2.37)^c^	9.97 (2.6)^d^	9.22 (2.63)^c^	167.59	*p* < 0.001	0.27
Remorselessness	8.66 (3.1)	13.12 (2.87)^a^	11.8 (2.59)^b^	7.84 (2.19)^c,d^	8.3 (2.55)^d^	7.68 (2.46)^c^	234.25	*p* < 0.001	0.34
Unemotionality	10.45 (3.08)	14.65 (2.47)^a^	13.34 (2.15)^b^	9.15 (1.93)^c^	9.75 (2.38)^c^	9.79 (2.81)^c^	193.08	*p* < 0.001	0.3
Anxiety	2.63 (3.1)	1.66 (1.79)^a,c^	8.25 (1.94)^b^	8.68 (2.16)^b^	2.03 (1.87)^a^	1.63 (2.27)^c^	532.03	*p* < 0.001	0.54
Maltreatment	*n* = 668	*n* = 62	*n* = 42	*n* = 65	*n* = 137	*n* = 362			
Physical abuse	5.9 (2.44)	6.6 (3.08)^a^	7.4 (3.48)^a^	6.75 (3.66)^a^	6.39 (3.17)^a^	5.26 (0.92)^b^	13.12	*p* < 0.001	0.07
Sexual abuse	5.55 (2.5)	5.5 (2.22)^a^	5.69 (2.1)^a^	6.88 (4.73)^a^	6.01 (3.46)^a^	5.12 (1.1)^b^	7.01	*p* < 0.001	0.04
Emotional abuse	7.52 (3.63)^a^	8.77 (4.88)^a^	9.57 (4.29)^a^	10.25 (5.25)^a^	8.03 (3.61)^a^	6.38 (2.23)^b^	30.78	*p* < 0.001	0.16
Physical neglect	6.69 (2.47)	7.58 (2.85)^a^	7.93 (2.87)^a^	7.54 (3.05)^a^	7.19 (3.01)^a^	6.05 (1.71)^b^	11.98	*p* < 0.001	0.07
Emotional neglect	9.44 (4.47)	11.89 (5.78)^a^	11.83 (4.97)^a^	11.08 (4.54)^a^	10.77 (4.81)^a^	7.95 (3.32)^b^	21.01	*p* < 0.001	0.11
Parenting	*n* = 1548	*n* = 155	*n* = 86	*n* = 108	*n* = 331	*n* = 868			
Parental involvement	34.32 (6.22)	30.85 (7.31)^a^	31.74 (7.09)^a^	33.04 (6.52)^a^	32.7 (7.03)^a^	35.98 (4.89)^b^	28.86	*p* < 0.001	0.07
Positive parenting	24.04 (4.34)	22.49 (5.15)^a^	23.13 (5.26)^a^	23.81 (4.34)^a,c^	23.18 (5.07)^a^	24.77 (3.58)^c^	15.58	*p* < 0.001	0.04
Poor supervision	23.52 (7.3)	29.08 (8)^a^	27.63 (7.29)^a^	24.26 (7.67)^b^	25.05 (7.59)^b^	21.44 (6.06)^c^	56.42	*p* < 0.001	0.13
Inconsistent discipline	15.43 (3.96)	17.52 (3.81)^a^	17.47 (3.39)^a^	17.18 (4.13)^a^	16.06 (4.46)^b^	14.4 (3.43)^c^	33.45	*p* < 0.001	0.08
Corporal punishment	4.26 (1.94)	5.09 (2.42)^a^	4.87 (2.49)^a^	4.46 (2.03)^a^	4.78 (2.41)^a^	3.82 (1.37)^b^	23.5	*p* < 0.001	0.06
YPI subscales									
Dishonest Charm	9.24 (3.45)	12.12 (3.62)^a^	11.74 (3.44)^a^	8.55 (3.15)^b,c^	9.23 (3.48)^b^	8.56 (3.03)^c^	59.69	*p* < 0.001	0.13
Grandiosity	8.66 (2.95)	10.83 (3.31)^a^	9.64 (3.18)^b^	7.77 (2.45)^c^	8.59 (2.88)^c^	8.3 (2.74)^d^	33.53	*p* < 0.001	0.08
Lying	8.57 (3.28)	10.83 (3.95)^a^	10.65 (3.52)^a^	8.92 (3.27)^b^	8.79 (3.39)^b^	7.8 (2.72)^c^	41.51	*p* < 0.001	0.09
Manipulation	8.52 (3.34)	11.59 (3.55)^a^	10.79 (3.7)^a^	7.94 (2.93)^b,c^	8.65 (3.44)^b^	7.75 (2.8)^c^	68.15	*p* < 0.001	0.14
Thrill seeking	12.96 (3.16)	15.88 (2.62)^a^	15.26 (2.7)^a^	13.23 (3.37)^b^	13.16 (3.17)^b^	12.07 (2.78)^c^	70.22	*p* < 0.001	0.15
Impulsiveness	12.09 (3.48)	14.91 (2.91)^a^	14.84 (2.53)^a^	13.66 (3.6)^b^	12.83 (3.68)^b^	10.78 (2.87)^c^	80.33	*p* < 0.001	0.17
Irresponsibility	9.26 (3.62)	12.97 (3.86)^a^	12.78 (3.39)^a^	10.17 (3.64)^b^	10.28 (3.7)^b^	7.69 (2.43)^c^	130.05	*p* < 0.001	0.24

*Note*: Group means with different superscript indices differed significantly in post hoc comparisons (*p* < 0.05, Bonferroni corrected).

Abbreviation: YPI, Youth Psychopathic traits Inventory.

TD youth and those in the primary CU trait and CD‐only subgroups were undifferentiated on levels of anxiety, and significantly lower than youth in the secondary CU trait and CD/Anx youth who did not differ from each other. Across all subgroups, there were no significant age differences, but TD youth were significantly higher than all other subgroups on measures of IQ and SES.

### Maltreatment

Youth in the primary and secondary subgroups were significantly higher than TD youth on physical and emotional abuse and neglect but were not significantly different from CD/Anx and CD‐only youth. Primary and secondary subgroups did not differ significantly from each other on any abuse or neglect measure (Table 3, Figure 1B).

For physical abuse, youth with primary (*M* = 6.6, *SD* = 3.08) and secondary (*M* = 7.4, *SD* = 3.48) CU traits fell into the minimal to moderate range. For sexual abuse, youth with primary (*M* = 5.5, *SD* = 2.22) and secondary (*M* = 5.69, *SD* = 2.1) CU traits fell into the moderate to severe range. For emotional abuse, youth with primary (*M* = 8.77, *SD* = 4.88) and secondary (*M* = 9.57, *SD* = 4.29) CU traits fell into the moderate to severe range. For physical neglect, youth with primary (*M* = 7.58, *SD* = 2.85) and secondary (*M* = 7.93, *SD* = 2.87) CU traits fell into the moderate to severe range. For emotional neglect, youth with primary (*M* = 11.89, *SD* = 5.78) and secondary (*M* = 11.83, *SD* = 4.97) CU traits fell into the moderate to severe range.

### Parenting

When compared with TD youth, primary and secondary subgroups had significantly higher experiences of poor parental supervision, inconsistent discipline, and corporal punishment and significantly lower experiences of parental involvement and positive parenting. Primary and secondary CU trait subgroups did not differ from each other on any parenting measure but had significantly higher experiences of poor parental supervision than CD/Anx and CD‐only youth and higher levels of inconsistent discipline than the CD‐only subgroup. Youth in the primary subgroup had significantly less parental involvement than CD/Anx and CD‐only youth (Table 3, Figure 1C).

### Sex differences

The 4‐class solution held equally well when tested separately in boys and girls. Further, the multi‐group model fit statistics improved from the configural (unconstrained) to the structural (constrained) model indicating the same number of profiles in boys and girls (Supplemental Table S2). Like the mixed sex results, the 4‐class solution in boys and girls had clearly identifiable subgroups of primary and secondary CU traits, CD/Anx, and CD‐only youth. Boys in the primary and secondary subgroups had significantly higher levels of CU traits than TD boys, and boys in the primary subgroup had significantly higher CU traits than boys in the secondary subgroup. Boys in the secondary subgroup had significantly higher levels of anxiety than boys in the primary subgroup and TD boys. Additionally, boys in primary and secondary subgroups were undifferentiated on levels of CD and significantly higher than TD boys. Girls in the primary and secondary subgroups had significantly higher levels of CU traits and CD than TD girls and were not significantly different from each other on the callousness subscale. However, girls in the primary subgroup were significantly higher than girls in the secondary subgroup on the remorselessness and unemotionality subscales. Girls in the secondary subgroup were significantly higher on anxiety levels than girls in the primary subgroup and TD girls (Supplemental Tables S3 and S4).

Boys in the primary and secondary subgroups were significantly higher than TD boys on emotional abuse. Boys in the primary subgroup had significantly more physical and emotional neglect than TD boys and boys in the secondary subgroup had significantly more physical abuse than TD boys. Primary and secondary subgroups did not differ from each other or the other CD subgroups on physical and emotional abuse and neglect. In the girls, primary and secondary subgroups were significantly higher than TD girls on measures of physical and emotional abuse and neglect but were not significantly different from each other or girls in the other CD subgroups on any measure of abuse or neglect (Supplemental Tables S3 and S4).

Boys with primary and secondary CU traits had significantly higher levels of poor parental supervision and inconsistent discipline, and significantly lower experiences of parental involvement than TD boys. Boys in the primary subgroup had significantly higher levels of corporal punishment than TD boys and significantly greater experiences of poor supervision than boys in the CD‐only subgroup. Girls in the primary and secondary subgroups had significantly greater levels of poor parental supervision, inconsistent discipline, and corporal punishment and significantly lower experiences of parental involvement and positive parenting than TD girls, significantly higher experiences of poor supervision than girls in the other CD subgroups, and significantly higher levels of corporal punishment than girls in the CD‐only subgroup. Girls in the primary subgroup had significantly less parental involvement than girls in both CD subgroups (Supplemental Tables S3 and S4).

## DISCUSSION

This study, including a large mixed‐sex sample (*N* = 1831) with TD youth and youth with CD, contributes to our understanding of primary and secondary CU traits in four ways. First, we demonstrated that anxiety was the key feature distinguishing between primary and secondary CU trait subgroups by comparing LPA models using anxiety (i.e., a phenotypic feature), maltreatment (i.e., a putative risk factor), and a combination of both as continuous indicators. We showed that anxiety was the best classifier and that maltreatment without anxiety did not result in the same class membership. When anxiety and maltreatment were both included as classifiers, maltreatment did not aide in the classification of youth into primary and secondary subgroups as there was near perfect agreement between the anxiety‐only and the anxiety/maltreatment classifications and model fit statistics were poorer when maltreatment was included alongside anxiety.

Second, we found that youth in primary and secondary subgroups experienced greater levels of abuse and neglect when compared with TD youth but were not significantly different from each other or other CD subgroups on levels of abuse and neglect. Third, we found that youth with primary and secondary CU traits experienced significantly higher levels of maladaptive parenting and lower levels of positive parenting compared with TD youth. Primary and secondary CU trait subgroups did not differ from each other on any parenting measure but had significantly less parental supervision than youth in the CD subgroups and significantly higher experiences of inconsistent discipline than CD‐only youth. Youth in the primary CU trait subgroup had significantly less parental involvement than youth in other CD subgroups. Fourth, and crucially, we showed for the first time that anxiety can also be used to classify boys and girls into primary and secondary subgroups equally well. Additionally, sex differences were observed in maltreatment histories and parenting practices.

### Identifying primary and secondary subgroups of CU traits

To our knowledge, this is the first study to test how different indicators (i.e., anxiety, maltreatment, or both) for primary and secondary CU traits influence the number and nature of classes obtained in a large, mixed‐sex sample of youth with CD and TD youth. Consistent with our predictions, our findings suggest that classification of primary and secondary CU traits varies according to whether anxiety (i.e., a phenotypic feature) or maltreatment (i.e., a putative risk factor) are used as indicators. Specifically, the best fitting model included CU traits and anxiety as indicators and yielded a four‐class solution, which was interpretable with identifiable primary and secondary CU subgroups and two CD subgroups with low levels of CU traits, but high and low levels of anxiety. The proportion of youth assigned to the primary and secondary subgroups was consistent with previous work (Craig, Goulter, & Moretti, 2021) with the primary subgroup being the largest of the two. Importantly, these four subgroups were also identified when boys and girls were analysed separately indicating that anxiety consistently disaggregated subgroups of CU traits and CD‐only youth across sexes. Clearly defined, theoretically interpretable subgroups were not evident when CU traits and maltreatment were used as indicators. Instead, the subgroups only differed by CU traits. Furthermore, when classified using CU traits and maltreatment, participants were not significantly different from each other on anxiety levels. This suggests that maltreatment‐derived primary and secondary subgroups can lead to groups that are heterogenous in anxiety. Moreover, when compared with CU traits and anxiety, using maltreatment alongside anxiety as an indicator did not aid the classification of youth into primary and secondary subgroups, with near identical classifications with the anxiety‐only solution and poorer model fit indices.

Our mixed and same‐sex (discussed below) findings support the hypothesis (e.g., Craig, Goulter, & Moretti, 2021; Karpman, 1941) that anxiety is a key distinguishing feature between primary and secondary CU traits. The different group allocations across the LPA models suggest that anxiety and maltreatment do not identify the same subgroups of participants when used independently. These findings strengthen the views that (i) putative risk factors such as maltreatment should not be included with symptoms (i.e., anxiety) when defining primary and secondary CU traits and ii) a unified approach for identifying primary and secondary CU traits must be adopted (Todorov et al., 2023) to support cumulative science aiming to identify different etiological pathways and treatment needs.

### CU subgroups and maltreatment

Consistent with our hypothesis, both primary and secondary CU trait subgroups were more likely to have maltreatment histories (i.e., abuse and/or neglect) than TD youth and equally as likely as youth with CD and low CU traits. In previous studies using anxiety as a continuous indicator, primary and secondary subgroups, compared to youth with conduct problems but without CU traits, demonstrated either equivalent experiences of maltreatment (Kimonis et al., 2017; Rosan et al., 2015) or higher experiences of maltreatment for the secondary subgroup (Euler et al., 2015; Kimonis et al., 2012). While most studies associate the secondary subgroup with maltreatment, primary subgroups have also demonstrated maltreatment histories when compared with incarcerated youth (Euler et al., 2015; Kimonis et al., 2012; Sharf et al., 2014). There is a paucity of studies comparing the maltreatment histories of youth with primary and secondary CU traits to TD youth. Thus, the absence of TD youth in studies that have included maltreatment measures may obscure the specific maltreatment histories in primary subgroups. In a recent review of research on primary and secondary CU traits (Craig, Goulter, & Moretti, 2021) none of the studies that included TD youth also included maltreatment measures, which makes the comparison with our results difficult. Our findings suggest that youth in the primary subgroup have experienced greater levels of maltreatment than TD youth, which highlights the importance of abuse and neglect histories for youth in the primary subgroup as much as for youth in the secondary subgroup. This is in line with what Karpman (1941, 1956) suggested but remains obscured in the field with existing study designs (e.g., comparing the primary subgroup to CD‐only youth who have high maltreatment histories).

Contrary to our predictions and previous findings (e.g., Kimonis et al., 2013), no significant differences in abuse and neglect were observed between youth in primary and secondary CU subgroups. Here again, it is difficult to compare our study with previous ones because the majority of studies have either measured abuse only or combined abuse and neglect into a single risk factor (Craig, Goulter, & Moretti, 2021) making it impossible to disentangle unique effects (see Todorov et al., 2023) and potentially obscuring associations with the primary subgroup (Graham et al., 2012). Broader research suggests that neglect might be more pervasive and severe than abuse (Bland et al., 2018; Braga et al., 2018) with both overlapping (Haahr‐Pedersen et al., 2020) and distinct (Buisman et al., 2019) developmental consequences. While combined abuse and neglect contribute to internalizing and externalizing behaviours, participants with histories of neglect have been found to have the highest levels of externalizing problems (Vachon et al., 2015) and impaired empathy (Zhang et al., 2022). Psychopathic features in adults have been linked with reduced or absent affective experiences in childhood (Bisby et al., 2017; Cleckley, 1982) and, while abuse histories have been mainly theorised in relation to secondary subgroups, parental rejection and neglect may also play a key role in the development of the primary subgroup (Karpman, 1956). The impact of neglect is likely masked by abuse comorbidity (Bland et al., 2018) and the combination of abuse and neglect as one category of maltreatment (Todorov et al., 2023). Further, given that secure attachment requires the absence of neglect through appropriate parental responsiveness (Lyons‐Ruth & Jacobvitz, 1999) and that youth with elevated CU traits and adults with psychopathy are more likely to exhibit disorganized attachment patterns (De Brito et al., 2021), it is likely that neglect plays a far greater role than is currently recognised. Indeed, researchers in attachment have long noted that disorganized attachment patterns found in maltreated children may stem from inconsistent care (Carlson et al., 1989; Tyler et al., 2006). In this context, while abuse has received the majority of attention in CU trait/psychopathy studies, neglect, an equally costly cause of sorrow, has not been measured by its worth (Shakespeare, 1606/2015). Our findings and previous work (e.g., Kimonis et al., 2013) suggest the importance of neglect as separate form of maltreatment, alongside abuse. Taken together, these data provide further support to the view that defining CU subgroups using putative causal risk factors such as maltreatment may confound results by obscuring the presence of maltreatment histories in the subgroup with primary CU traits (Todorov et al., 2023). To understand the extent to which maltreatment subtypes represent causal risk factors, longitudinal and genetically informed research designs are needed to investigate subtypes of maltreatment within subgroups of youth with primary and secondary CU traits defined by anxiety levels.

While our findings are in line with previous work showing that neglect and abuse often co‐occur (Haahr‐Pedersen et al., 2020), the maltreatment subtypes were correlated but not completely overlapping (Supplemental Table S1) and similar to a recent meta‐analysis of primary and secondary CU traits and maltreatment histories (Todorov et al., 2023). In sum, our findings provide support for the claim that abuse and neglect subtypes of maltreatment should both be investigated within studies, but as separate constructs.

### CU subgroups and maladaptive parenting practices

Consistent with our hypothesis, youth with primary and secondary CU traits had less positive parenting and parental involvement and more experiences of poor supervision, inconsistent discipline, and corporal punishment than TD youth. When compared to CD subgroups, primary and secondary subgroups had significantly higher experiences of poor parental supervision than both CD subgroups, and higher levels of inconsistent discipline than the CD/Anx subgroup. Youth with primary and secondary CU traits did not differ from each other, but youth in the primary subgroup had significantly less parental involvement than youth in both CD subgroups. Given the high heritability of CU traits (Pezzoli et al., 2024), and evidence that warm, positive parenting buffers genetic risk for CU traits (Hyde et al., 2016; Waller et al., 2016), it is surprising that so little research has been dedicated to examining these parenting factors in relation to primary and secondary CU traits. This gap is noteworthy considering different causal factors and responses to treatment, higher risk for more significant impairment, and inconsistent findings with respect to parenting practices in research (Frick, 2024; Pezzoli et al., 2024). The few existing studies have found that both CU trait subgroups were higher than TD youth, but not significantly different from each other, on harsh parenting (Ezpeleta et al., 2017; Humayun et al., 2014; Meehan et al., 2017), that neither subgroup experienced harsh parenting (Craig, Goulter, Andrade, & McMahon, 2021), or that the secondary subgroup were more likely to experience harsh parenting (Goulter et al., 2017). To our knowledge, this is the first study to investigate parental involvement and positive parenting measures with respect to primary and secondary subgroups of CU traits. The findings warrant further investigation into Karpman's (1956) theory that primary variants may be characterised by neglect and parental rejection.

Maltreatment subtypes and negative (*r* = 0.09–0.33) and positive (*r* = −0.12 to −0.41) parenting measures were correlated with each other, but not perfectly indicating overlapping but distinct constructs. While we did not find that primary and secondary subgroups of CU traits differed on any maltreatment subtype or parenting measure, this may not be the case with younger samples and different methods. For example, prospective longitudinal studies involving direct observation of parent‐child interaction early in development (e.g., Fleming et al., 2022) could be very useful. Clearly, replications of our findings using a uniform approach to identify primary and secondary subgroups is needed to understand the role of parenting in primary and secondary subgroups of CU traits.

### Sex differences

In both boys and girls, results were similar to the findings for the overall sample with clearly identifiable subgroups of youth with primary and secondary CU traits, CD/Anx and CD‐only. Boys with primary CU traits had significantly higher levels of CU traits than boys in the secondary subgroup. Youth in primary and secondary CU trait subgroups were equally high on levels of CD and significantly higher than the other subgroups, with the exception of the CD/Anx subgroup, which was not significantly different from the high CU trait subgroups on levels of CD. Youth in the secondary CU trait and CD/Anx subgroups were significantly higher than all other groups on levels of anxiety. In girls, similar results were observed.

When boys and girls were analysed separately on maltreatment measures, results in girls were consistent with the overall sample, but in boys, the primary subgroup in relation to physical abuse and the secondary subgroup in relation to physical and emotional neglect did not differ significantly when compared with TD youth. This could reflect sex differences in abuse and neglect histories of youth with primary and secondary CU traits. However, it could also reflect a loss of statistical power in subgroups of primary (*n* = 46) and secondary (*n* = 19) boys with CU traits. The latter seems the more likely explanation as boys in primary and secondary CU trait subgroups were not significantly different from each other on any abuse or neglect measure. Finally, it is difficult to parse the role of neglect because the majority of studies to date have combined abuse and neglect together into one category (Craig, Goulter, & Moretti, 2021). Replication is needed to confirm the findings of the current study.

When parenting practices were analysed separately by sex, boys with primary and secondary CU traits had significantly higher experiences of poor parental supervision and inconsistent discipline, and significantly lower experiences of parental involvement than TD boys. Boys in the primary subgroup had significantly higher experiences corporal punishment than TD youth and significantly higher experiences of poor supervision than boys in the CD‐only subgroup. Girls in the primary and secondary subgroups had significantly higher experiences of poor parental supervision, inconsistent discipline, and corporal punishment and significantly lower experiences of parental involvement and positive parenting than TD girls. When compared with girls in the CD/Anx and CD‐only subgroups, girls in the primary and secondary subgroups had significantly higher experiences of poor supervision than both and significantly higher levels of corporal punishment than girls in the CD‐only subgroup. Girls in the primary subgroup had significantly less parental involvement than girls in the CD/Anx and CD‐only subgroups (Supplemental Tables S3 and S4). These findings contrast with an all‐female study where the secondary subgroup had higher experiences of negative parenting than the primary subgroup (Goulter et al., 2017) and suggest potential sex differences between the subgroups. However, there are few studies to date with combined investigation into primary and secondary subgroups, parenting practices, and sex differences. Genetically informed, prospective longitudinal research is needed to ascertain what sex differences may be present.

### Clinical implications

Defining primary and secondary subgroups of CU traits by anxiety, understanding risk factors of maltreatment and maladaptive parenting for *both* subgroups relative to anxiety levels, and considering sex differences all have important implications for assessment and treatment planning. For example, with respect to the Limited Prosocial Emotions specifier in the DSM, routine assessment of anxiety could be important because youth with CD may have high or low anxiety and those with CD and high CU traits could fall into primary or secondary subgroups that are potentially relevant for trajectory, prognosis, and treatment needs (Fanti, 2018; Fanti & Kimonis, 2017). There is also evidence for different trajectories of conduct problems in youth with primary and secondary CU traits, which increase or decrease over time and may have different risk processes and treatment needs (Ezpeleta et al., 2017). Further, putative causal risk factors of maltreatment and maladaptive parenting appear to be present in youth with high and low anxiety suggesting these risk factors are relevant for both subgroups and subsequent treatment planning. And finally, since anxiety has a high rate of comorbidity and is more prevalent in girls with more severe symptoms that manifest differently than for boys (Farhane‐Medina et al., 2022), there is a need to consider sex differences in assessment and treatment planning/intervention efforts.

### Strengths and limitations

Some limitations are worth noting. First, while the process of classification does not hinge on causal inferences, future research using longitudinal and genetically informed designs is needed to clarify the developmental processes giving rise to primary and secondary CU traits. Second, in line with the original aims of the FemNAT‐CD study, there was an oversampling of female participants, which could contribute to a loss of power in findings for boys (Freitag et al., 2018). However, past research has been largely conducted in all‐male samples, making the contribution of a study where girls are in the majority a positive contribution to existing work. Third, measures for maltreatment subtypes relied upon retrospective, self‐report measures, and meta‐analytic work has shown that retrospective reports and prospective measures may not identify the same populations (Baldwin et al., 2019; Negriff et al., 2017). However, it is also worth noting that recent meta‐analytic evidence suggests that the effects of early adversity on psychopathology are predominantly driven by subjective experience (Francis et al., 2023). Additionally, though we use 9–18‐year age range with measures for both child and parent, the absence of younger aged children may obscure parenting effects that are most influential in younger children. Despite these limitations, several strengths of this study should be noted including the large, mixed‐sex sample, the inclusion of TD youth which allowed for detailed comparison of group differences, the use of robust clinical interview to assess CD and anxiety, the data‐driven approach using LPA to identify subgroups, the use of multiple informants, and rigorous semi‐structured interviews for measuring psychopathology in all the participants.

## CONCLUSION

Our findings suggest that anxiety and maltreatment cannot be used interchangeably to identify youth with primary versus secondary CU traits. Anxiety yielded the best fitting and most theoretically interpretable classifications *across both sexes*, supporting the view that anxiety is the distinguishing feature between primary and secondary CU subgroups. We propose that anxiety should be used to define CU subgroups instead of a putative causal risk factor such as maltreatment since both primary and secondary subgroups are associated with childhood maltreatment. Combining abuse and neglect into overall maltreatment measures may obscure distinct maltreatment subtype associations in youth with primary CU traits or between the CU trait subgroups. The results signal the need for clinical and operational guidelines on how to define primary and secondary subgroups of CU traits. Patterns of association between CU trait subgroups and maltreatment histories varied across sexes suggesting sex differences between primary and secondary subgroups of CU traits. Additionally, beyond maltreatment, primary and secondary CU subgroups were characterised by maladaptive parenting experiences. Prospective, longitudinal research is needed to elucidate causal mechanisms in addition to developing a consistent and uniform method for defining primary and secondary subgroups of CU traits.

## AUTHOR CONTRIBUTIONS

**Jessica J. Todorov**: Conceptualization; formal analysis; methodology; software; writing – original draft; writing – review & editing. **Gregor Kohls**: Conceptualization; data curation; formal analysis; investigation; methodology; project administration; supervision; visualization; writing ‐ review & editing. **Ruth Pauli**: Data curation; methodology; writing ‐ review & editing. **Jack Rogers**: Data curation; methodology; writing ‐ review & editing. **Anka Bernhard**: Data curation; methodology; writing ‐ review & editing. **Katharina Ackermann**: Data curation; methodology; writing ‐ review & editing. **Nora M. Raschle**: Data curation; methodology; writing ‐ review & editing. **Jules R. Dugre**: Formal analysis; methodology; visualization; writing – review & editing. **Aranzazu Fernandez‐Rivas**: Conceptualization; investigation; methodology; project administration; supervision; writing ‐ review & editing. **Miguel Angel Gonzalez‐Torres**: Investigation; methodology; project administration; supervision; writing ‐ review & editing. **Amaia Hervas**: Conceptualization; investigation; methodology; project administration; supervision; writing ‐ review & editing. **Areti Smaragdi**: Data curation; methodology; writing ‐ review & editing. **Karen Gonzalez**: Data curation; methodology; writing ‐ review & editing. **Agnes Vetro**: Data curation; methodology; writing ‐ review & editing. **Dimitris Dikeos**: Conceptualization; investigation; methodology; project administration; supervision; writing ‐ review & editing. **Arne Popma**: Conceptualization; data curation; methodology; investigation; project administration; writing ‐ review & editing. **Christina Stadler**: Conceptualization; data curation; methodology; investigation; project administration; writing ‐ review & editing. **Kerstin Konrad**: Conceptualization; data curation; methodology; investigation; project administration; writing ‐ review & editing. **Christine M. Freitag**: Conceptualization; data curation; investigation; methodology; project administration; supervision; visualization; writing ‐ review & editing. **Graeme Fairchild**: Conceptualization; data curation; investigation; methodology; project administration; supervision; visualization; writing ‐ review & editing.  **Rory T. Devine**: Conceptualization; data curation; formal analysis; investigation; methodology; project administration; supervision; visualization; writing ‐ original draft; writing – review & editing. **Stephane A. De Brito**: Conceptualization; data curation; formal analysis; investigation; methodology; project administration; supervision; visualization; writing ‐ original draft; writing – review & editing.

## CONFLICT OF INTEREST STATEMENT

Christina Stadler receives royalties for books on ODD/CD, Christine M. Freitag receives royalties for books on ASD and ADHD.

## ETHICAL CONSIDERATIONS

Ethical committees at each site approved the study. Ethical approval details for the sites are provided in Supplemental S1.

## Supporting information

Supporting Information S1

## Data Availability

The data presented in this article is not openly available but are available on reasonable request in line with the consortiums' data sharing policy. Requests to access the data should be directed to Stephane De Brito: s.a.debrito@bham.ac.uk.
