# Supplementary material for: Maltreatment and parenting in youth with primary and secondary callous‐unemotional traits: Anxiety matters
Source: JCPP Adv. 2024 Jul 30;5(2):e12266. doi: 10.1002/jcv2.12266 (PMC12159302; doi:10.1002/jcv2.12266)

**Supplementary Material**

**Maltreatment and parenting in youth with primary and secondary callous-unemotional traits: Anxiety matters.**

Jessica J. Todorov^1^, Gregor Kohls^3^, Ruth Pauli^1^, Jack Rogers^4^, Anka Bernhard^10,^ Katharina Ackermann^10^, Nora Raschle^15^, Jules R. Dugre^1^, Aranzazu Fernandez-Rivas^5^, Miguel Angel Gonzalez-Torres^5^, Amaia Hervas^12^, Areti Smaragdi^9^, Karen Gonzalez^9^, Ágnes Vetró^14^, Dimitris Dikeos^11^, Arne Popma^13^, Christina Stadler^6^, Kerstin Konrad^7,8^, Christine M. Freitag^4^, Graeme Fairchild^9^, Rory T. Devine^2*^ Stephane A. De Brito^1*^

## S1 Ethical Approval

The FemNAT-CD project received ethical approval from the following relevant local ethics committees:

Aachen: Ethik Kommission Medizinische Fakultät der Rheinisch Westfälischen Technischen Hochschule Aachen (EK027/14). Amsterdam: Medisch Etische Toetsingscommissie (2014.188). Athens: Election Committee of the First Department of Psychiatry, Eginition University Hospital (641/9.11.2015). Barcelona: Child and Adolescent Mental Health - University Hospital Mutua Terrassa (acta 12/13). Basel: Ethik Kommission Nordwest- und Zentralschweiz (EKNZ 336/13). Bilbao: Hospital del Basurto. Birmingham and Southampton: University Ethics Committee and National Health Service Research Ethics Committee (NRES Committee West Midlands, Edgbaston; REC reference 3/WM/0483). Dublin: SJH/AMNCH Research Ethics Committee (2014/04/Chairman (3)). Frankfurt: Ethik Kommission Medizinische Fakultät Goethe Universität Frankfurt am Main (445/13). Szeged (Hungary): Egészségügyi Tudományos Tanács Humán Reprodukciós Bizottság (CSR/039/00392-3/2014).

**SUPPLEMENTARY MATERIAL METHODS**


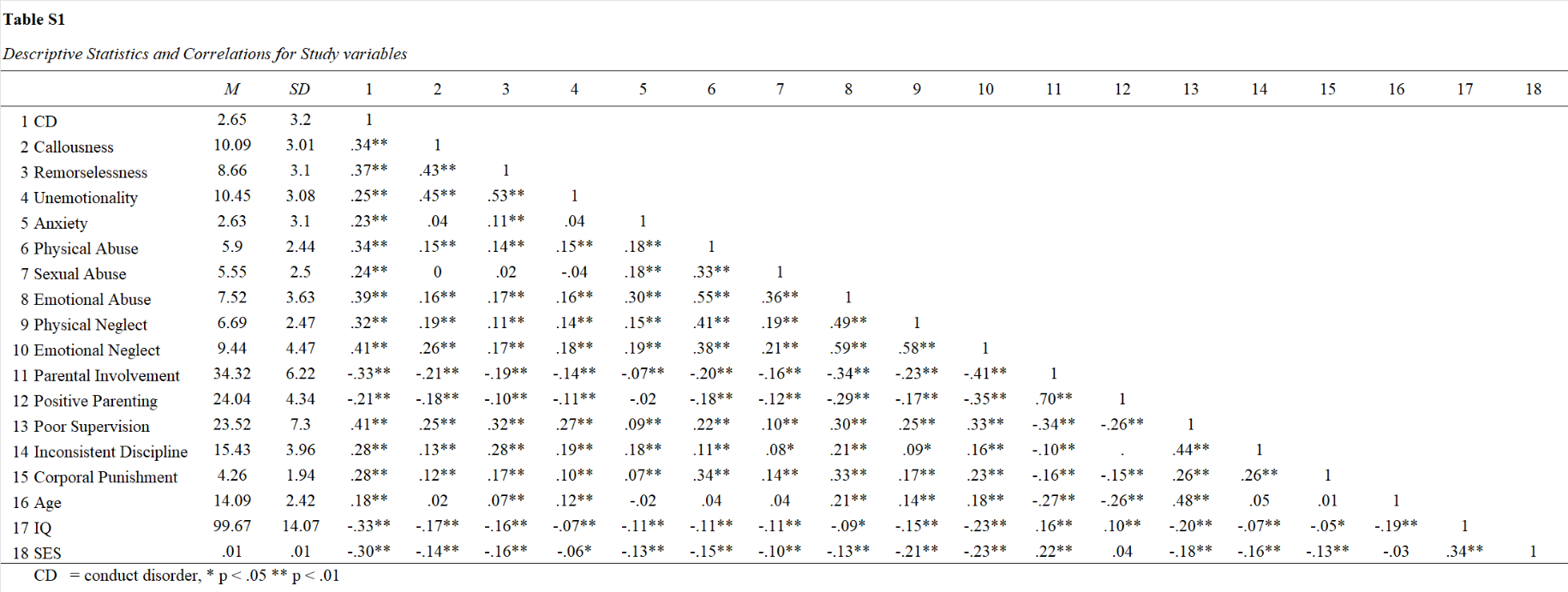


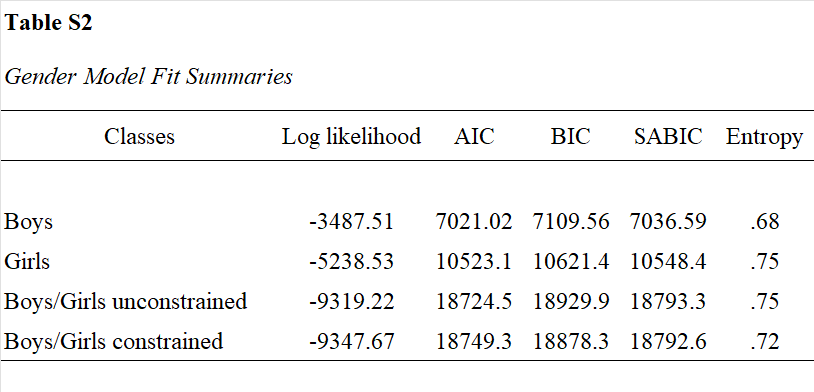


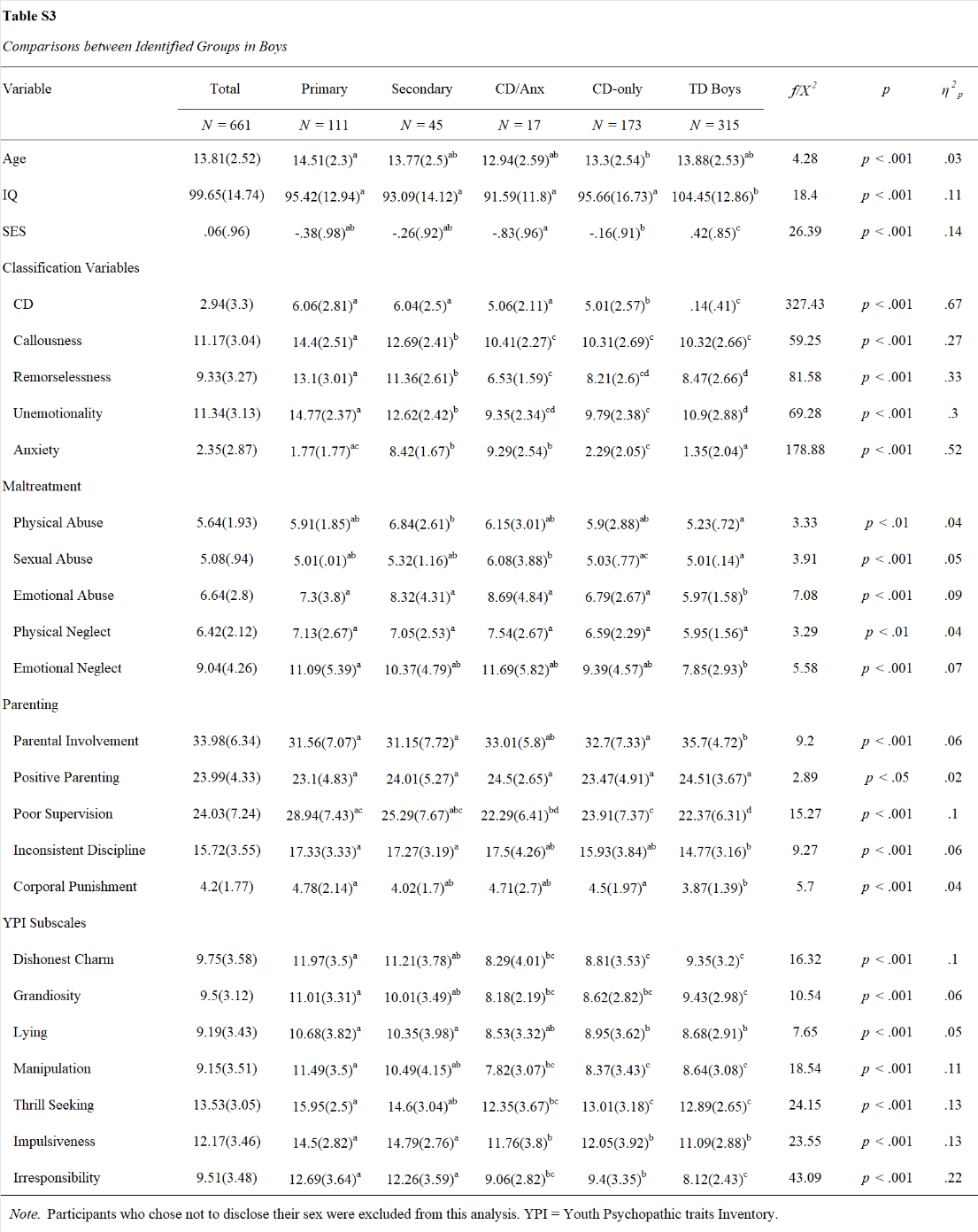


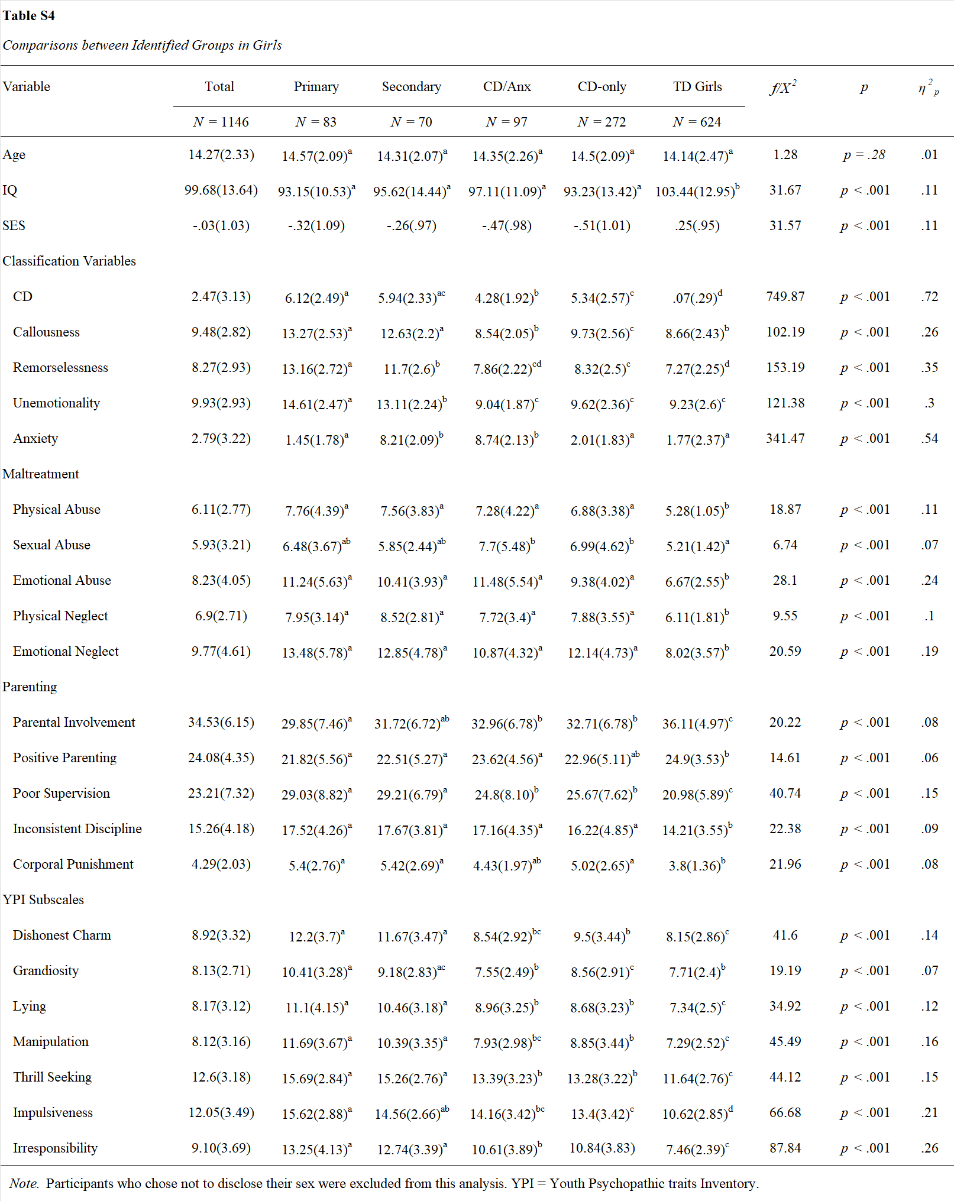

Supplement: Supplementary file 1 — Supporting Information S1 [file JCV2-5-e12266-s001.docx]
